# Molecular Characterization of *Trypanosoma evansi* Mevalonate Kinase (TeMVK)

**DOI:** 10.3389/fcimb.2018.00223

**Published:** 2018-07-10

**Authors:** Daniel P. Duarte, Éden R. Ferreira, Fabio M. Lima, Franciane Batista, Michel De Groote, Eduardo Horjales, Luiz C. Miletti, Diana Bahia

**Affiliations:** ^1^Laboratório de Bioquímica de Hemoparasitas e Vetores-Centro de Ciências Agroveterinárias, Universidade do Estado de Santa Catarina, Lages, Brazil; ^2^Departamento de Microbiologia, Imunologia e Parasitologia, Escola Paulista de Medicina, Universidade Federal de São Paulo, São Paulo, Brazil; ^3^Centro Universitário São Camilo, Avenida Nazaré, São Paulo, Brazil; ^4^Instituto de Física de São Carlos, Universidade de São Paulo, São Carlos, Brazil; ^5^Departamento de Biologia Geral, Instituto de Ciências Biológicas, Universidade Federal de Minas Gerais, Belo Horizonte, Brazil

**Keywords:** *Trypanosoma evansi*, mevalonate kinase, Surra, 3-hydroxy-3-methylglutaryl-CoA reductase, enzyme activity

## Abstract

The mevalonate pathway is an essential part of isoprenoid biosynthesis leading to production of a diverse class of >30,000 biomolecules including cholesterol, heme, and all steroid hormones. In trypanosomatids, the mevalonate pathway also generates dolichols, which play an essential role in construction of glycosylphosphatidylinositol (GPI) molecules that anchor variable surface proteins (VSGs) to the plasma membrane. Isoprenoid biosynthesis involves one of the most highly regulated enzymes in nature, 3-hydroxy-3-methylglutaryl-CoA reductase (HMGCR), which catalyzes the conversion of HMG-CoA to mevalonic acid. The enzyme mevalonate kinase (MVK) subsequently converts mevalonic acid to 5-phosphomevalonic acid. *Trypanosoma evansi* is a flagellate protozoan parasite that causes the disease “Surra” in domesticated large mammals, with great economic impact. *T. evansi* has only a trypomastigote bloodstream form and requires constant modification of the variant surface glycoprotein (VSG) coat for protection against the host immune system. We identified MVK of *T. evansi* (termed TeMVK) and performed a preliminary characterization at molecular, biochemical, and cellular levels. TeMVK from parasite extract displayed molecular weight ~36 kDa, colocalized with aldolase (a glycosomal marker enzyme) in glycosomes, and is structurally similar to *Leishmania major* MVK. Interestingly, the active form of TeMVK is the tetrameric oligomer form, in contrast to other MVKs in which the dimeric form is active. Despite lacking organized mitochondria, *T. evansi* synthesizes both HMGCR transcripts and protein. Both MVK and HMGCR are expressed in *T. evansi* during the course of infection in animals, and therefore are potential targets for therapeutic drug design.

## Introduction

*Trypanosoma evansi* (Kinetoplastida: Trypanosomatidae) is a widely distributed hemoflagellate protozoan parasite that causes the disease “Surra” in domesticated large mammals (e.g., horses, cattle, camels, water buffalo) and has major epidemiologic and economic impacts (Desquesnes et al., 2013a). *T. evansi* infection is included in the list of notifiable diseases by the World Organization for Animal Health (OIE; originally called Office International des Epizooties). Transmission of *T. evansi* is exclusively mechanical, predominantly by biting hematophagous flies (Silva et al., 2002). The species has spread far beyond its primary territory (sub-Saharan Africa) and now affects livestock in Northern Africa, Asia, Central America, and South America. Surra is potentially fatal, particularly when left untreated in camels, horses, and dogs (Desquesnes et al., 2013b).

*T. evansi* is phylogenetically closely related to *T. brucei* (Carnes et al., 2015), but displays many differences on the subcellular level. All strains of *T. evansi* are either dyskinetoplastic (i.e., having kDNA without maxicircles or minicircles) (Schnaufer et al., 2002; Lai et al., 2008) or akinetoplastic (completely lacking kDNA) (Birhanu et al., 2016). The absence of functional mitochondria is presumably the reason why *T. evansi* is permanently locked in the bloodstream stage and relies on the glycolytic pathway as primary source of ATP (Timms et al., 2002). Glycolysis has been proposed to be the primary and possibly sole source of ATP in all bloodstream forms of African trypanosomes (Bringaud et al., 2006).

Trypanocidal drugs (e.g., diminazene aceturate) are clinically available, but development of drug resistance is often observed (Uilenberg, 1998; Tuntasuvan et al., 2003). Elucidation of essential metabolic pathways is crucial for identification of novel targets and development of specific therapies against diseases caused by African trypanosomes. Noteworthy, no effective protocol to maintain *T. evansi* isolates in axenic culture is available, which limits the execution and reproducibility of *in vitro* studies. Although many attempts have been made to cultivate different *T. evansi* isolates *in vitro* (Hirumi et al., 1997; Kumar et al., 2015; Birhanu et al., 2016), the parasite viability declines within few days and substantial molecular changes are introduced (e.g., complete loss of the kinetoplast). Alternatively, parasite propagation is achieved via experimentally infected animals (Hirumi et al., 1997).

Isoprenoid biosynthesis, an essential pathway in most eukaryotes, leads to production of cholesterol in mammals and ergosterol in fungi and parasites. The first step in the isoprenoid pathway is synthesis of 3-hydroxy-3-methylglutaryl-CoA (HMG)-CoA from acetyl-CoA, with acetoacetyl-CoA as intermediate. 3-hydroxy-3-methylglutaryl-CoA reductase (HMG-CoA reductase; HMGCR), one of the most highly regulated enzymes in nature, catalyzes the conversion of HMG-CoA to mevalonic acid. The subsequent essential enzyme of the isoprenoid pathway, mevalonate kinase (MVK), catalyzes phosphorylation of mevalonic acid to form mevalonate 5-phosphate (Coppens and Courtoy, 1996).

Subcellular localization, structural properties, and enzymatic activities of MVK have been described in the trypanosomes *T. brucei, Leishmania major*, and *T*. *cruzi*. Mevalonate phosphorylation is localized almost exclusively in glycosomes in *T. brucei* and *L. major* (Carrero-Lérida et al., 2009), whereas TcMVK is localized only partially in glycosomes in *T. cruzi* (Ferreira et al., 2016). Sgraja et al. (2007) previously reported the reactivity of MVK from *L. major*. In their study, no enzymatic activity was detected *in vitro* using monomeric forms of recombinant proteins from these species. Using a sensitive method, we recently demonstrated higher enzymatic activity of dimeric *T. cruzi* MVK, comparable to reported values in other species (Ferreira et al., 2016).

The cell surface of African trypanosomes is densely coated with ~10^7^ variant surface glycoprotein (VSG) molecules. These trypanosomes avoid host innate immune responses by undergoing antigenic variation through expression of structurally different forms of glycosylphosphatidylinositol (GPI)-anchored VSGs in a sequential manner. Although VSGs vary, the GPI core structure attached to the protein is constant. Biosynthesis of the core structure depends on dolichol production for synthesis of dolichol-dependent mannosyltransferase, provided by the mevalonate pathway (Smith and Bütikofer, 2010). The mevalonate pathway may be potentially explored as target candidate for drug design.

The mevalonate pathway in *T. evansi* has not been studied previously. We identified and characterized MVK from *T. evansi* (termed TeMVK) using recombinant proteins, and predicted its structure using homology modeling. The subcellular localization of TeMVK was elucidated. In contrast to MVK from *T. cruzi*, TeMVK was highly active in its tetrameric form. Expression of TeMVK and HMGCR proteins was detected in parasites isolated directly from experimentally infected animals. These findings demonstrate the presence of mevalonate pathway enzymes *in vivo* in *T. evansi* during the course of infection.

## Materials and methods

### Parasite cultures and strains

*T. brucei* strain 29–19 and *T. evansi* TeH (isolated from a horse in 2009 in the state of Rio Grande do Sul, southern Brazil) (Duarte et al., 2014) were used in this study. *T. brucei* procyclic forms were grown in SDM-79 medium supplemented with 10% fetal bovine serum at 25°C. Bloodstream forms of *T. brucei* were maintained at 37°C at 5% CO_2_ in HMI-9 medium supplemented with 10% fetal bovine serum and 10% Serum Plus (SAFC Biosciences). *T. evansi* bloodstream forms were obtained by experimental infection of non-immunosuppressed Wistar rats inoculated intraperitoneally with 10^6^ trypomastigotes. Parasitemia was estimated daily by microscopic examination of blood smears. When the parasitemia reached a peak of 1 × 10^6^ parasites/ml in the blood between 4 and 7 days post-infection, the animals were anesthetized with Zoletil (Fort Dodge) and blood containing the parasites was collected by cardiac puncture. After a Percoll (Sigma) gradient (Grab and Bwayo, 1982), the pre-cleared samples were purified on DEAE-cellulose column to the isolation of the parasites from blood cells (Lanham, 1968). The study was approved by the Animal Welfare Commitment—UDESC.

### Amplification, cloning, and sequencing

Total DNA from *T. evansi* (1 × 10^8^ cells) was extracted using the phenol-chloroform method. The putative MVK gene containing 990 bp was amplified using the primers 5′-GCGCGTCGACATGCACGTGGCTGTTAAGGAC-3′ and 5′-GGCCAGATCTATAGCTTACTTCCGCCGGGCTG-3′ in a PCR reaction containing 100 ng of the genomic DNA, 10 pmoles of each primer pair, 1U Pfu DNA polymerase with supplied enzyme buffer (Fermentas), 0.2 mM dNTPs and 25 mM MgSO_4_. Amplification was performed using a hot-start step of 5 min at 94°C, followed by 35 cycles of 30 s denaturation at 94°C, 30 s annealing at 67°C, 1 min extension at 72°C, followed by a final extension of 10 min at 72°C. The amplified fragment containing the TeMVK sequence was cloned in pGEM-T easy vector (Promega) and sequenced using dideoxynucleotide chain termination method with BigDye Terminator cycle sequencing chemistry on an ABI PRISM 3100 sequencer (Applied Biosystems). For recombinant protein expression, TeMVK sequence was cloned into the plasmid pQTEV (Addgene) with a N-terminal 6xHis-tag.

For *T. evansi* HMGCR, total RNA from *T. evansi, T. brucei* procyclic, and bloodstream forms (1 × 10^8^ cells) was extracted using TRIzol and treated with DNAse I (Invitrogen). First-strand cDNA was synthesized using ThermoScript rtPCR System according to the manufacturer's instructions (Invitrogen). DNAse I efficiency was assessed by conventional PCR of RNA samples used to synthesize cDNA using specific primers for tubulin gene. The HMGCR gene containing 128 bp was amplified using the primers 5′-CAAAGTGCGGAGGTGAAGA-3′ and 5′-CCAGCCATACCAAGAGGAAT-3′. Amplified gene fragments from *T. evansi* and *T. brucei* procyclic and bloodstream forms were cloned in pGEM-T easy vector system (Promega) and sequenced as described above for confirmation of specificity.

### Sequence alignment and modeling

Multiple protein sequence alignment of TeMVK with MVKs from trypanosomatids *T. brucei* MVK, TbMVK (Q581L1, UniProt) *T. cruzi* MVK, TcMVK (Q4DJT8) and *L. major* MVK, LmMVK (Q4Q6K7) was generated using Geneious 9.1.5. The primary sequence of TeMVK was used for secondary structure analysis and homology modeling. TeMVK secondary structure was estimated *ab initio* using Jpred4 (Jared 4) prediction tool. The homology model of TeMVK was built based on two crystal structures of LmMVK (2HFU and 2HFS). TeMVK was modeled using the software Jackal 1.5 and submitted to a molecular dynamic *in silico* using the extension NAMD 2.1 in the software VMD 1.9.3. The molecular dynamic was done by first adding hydrogens to the protein structure and subsequently adding water molecules and sodium and chloride atoms for charge neutralization. The model was stabilized by a step of protein-energy minimization followed by total system energy minimization. The dynamic was performed during ~40 ns at 298 K. Model stability was assessed by the root-mean-square deviation (RMSD) of atomic positions values with standard deviation (SD) in angstroms (Å) for the backbone and carbon-α chain. The stereochemical quality of the backbone was verified using RAMPAGE tool (Lovell et al., 2003). Images were generated using Yasara v16.7.22.

### Protein expression and purification

The N-terminal 6xHis-tagged TeMVK was recombinantly expressed in *E. coli* BL21 (DE3). Cells were grown in autoinduction medium ZYM5052 at 20°C, 150 RPM during 45 h (Studier, 2005). For total cell lysate, cells were re-suspended in buffer containing 150 mM Tris-HCl pH 7.8, 300 mM NaCl, 5% glycerol and 0.1 mg/mL of lysozyme, followed by incubation in ice bath shaking for 40 min. Next, cells were disrupted by sonication during 15 min in cycles 30 s on/30 s off on ice. After addition of 1 μL of Benzonase (Sigma) and incubation 1 h at 4°C, the lysate was clarified by centrifugation for 10 min at 20,100 × g at 4°C. Five millimolars β-mercaptoethanol was added to the clarified lysate.

Protein expression with the expected molecular weight was verified by denaturing polyacrylamide gel electrophoresis (SDS-PAGE) stained with Coomassie Brillant Blue. Protein purification was done in two steps. First, total cell lysate was applied to a column containing 1 mL of Ni-NTA Superflow resin (Qiagen) coupled to a high-pressure chromatography system (AKTA) for affinity chromatography. Protein was eluted from the nickel column with ~200 mM of imidazole using an increasing gradient of buffer containing 150 mM Tris pH 7.8, 300 mM NaCl, 5% glycerol, and 500 mM imidazole. The second purification step performed was a size exclusion chromatography through a column Superdex 200 16/600 using buffer 30 mM glycine pH 9.0 and 150 mM NaCl. Gel filtration standard (Bio-rad) calibration of the columns indicates an approximated estimation of molecular weight. Fractions containing TeMVK were used for subsequent analysis or directly frozen at −80°C.

### Activity assay

Mevalonate kinase activity was measured as previously described (Ferreira et al., 2016). Briefly, recombinant TeMVK was tested to specific activity using monomeric, dimeric and tetrameric oligomers obtained from size exclusion chromatography in a reaction buffer containing 100 mM glycine, 25 mM KCl pH 9.0, 4 U of lactic dehydrogenase, 4 U of pyruvate kinase, 1 mM phosphoenolpyruvate, 5 mM ATP, 5 mM MgCl_2_, 4 mM mevalonate, and 160 μM β-NADH. The assays were done in triplicates in 96 well plates, and fluorescence at 340 ηm monitored using a Spectramax-340PLUS. Two concentrations of TeMVK (48 ηM and 156 μM) were used to verify NADH oxidation within 10 min at 37°C. Blank assay was performed in the absence of ATP. Independent protein production and purification were done for each assay. Results are reported as mean ± standard deviation (SD) and data analyzed using GraphPad Prism.

### Cell lysis and western blotting

Protein extracts from isolated parasites were obtained using urea-thiourea lysis buffer (7.7M urea, 2.2M thiourea, 4.4% CHAPS). Approximately 30 μg of total protein extracts from procyclic and bloodstream forms of *T. brucei* and *T. evansi* were resolved in a 10% SDS-PAGE, transferred to nitrocellulose membrane and blocked with 5% of non-fat dry milk (Cell Signaling) diluted in TBS 0.1% Tween-20 (TBST). For MVK detection, the membrane was incubated 12 h at 4°C with a polyclonal antibody specific to *T. cruzi* mevalonate kinase raised in mouse (Ferreira et al., 2016). Next, the membrane was washed with TBST and incubated with anti-mouse peroxidase for 1 h at room temperature. Signal detection was subsequently done by incubation with chemiluminescence reagent (GE Healthcare, cat. no: RPN2232) and detection using x-ray films. Western blot quantification was performed with ImageJ. An anti-human HMGCR antibody (Santa Cruz) was used for protein detection using similar protocol as described above.

### Immunofluorescence assay

Double staining immunofluorescence assays were performed using anti-*T. cruzi* mevalonate kinase, TcMVK (Ferreira et al., 2016) raised in mouse diluted 1:100 and the glycosomal marker anti-*L. major* aldolase raised in rabbit diluted 1:400. First, parasites were fixed with PBS 0.5% paraformaldehyde solution. After three washes, parasites were incubated with blocking/permeabilization solution (0.2% gelatin, 0.1% NaN_3_, and 0.1% saponin diluted in PBS) followed by 4 h primary antibodies incubation (4 h for each primary antibody). Secondary antibodies used were conjugated anti-rabbit Alexa-568 and anti-mouse Alexa-488 (Life Technologies) diluted 1:300. Nuclear and kinetoplast staining was performed using 4′, 6-diamidino-2-phenylindole (DAPI). Images were acquired using confocal microscope Leica SP5, and IMARIS software (Bitplane) was used to perform reconstruction and colocalization analysis.

## Results

### TeMVK is structurally similar to MVK from other trypanosomatids

**Figure 1:**
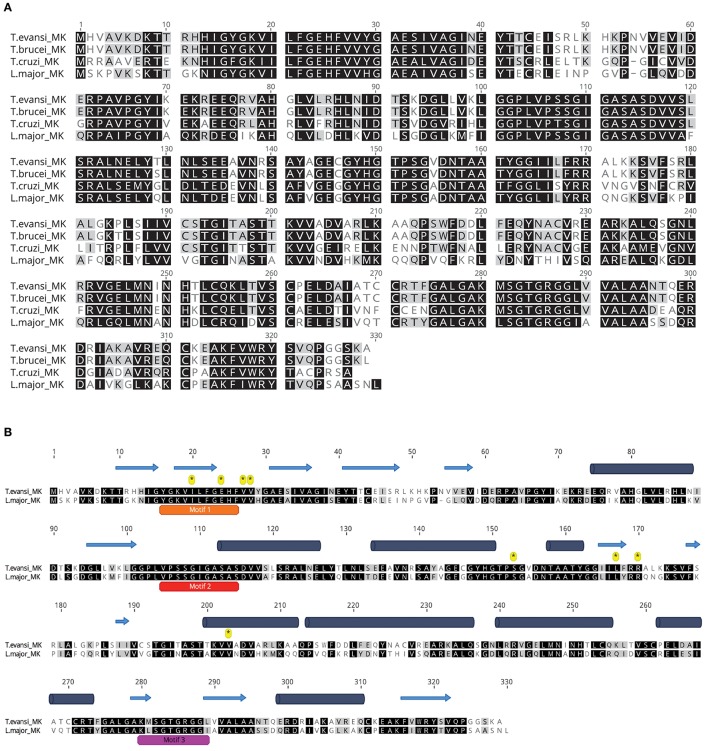
TeMVK is highly similar to MVKs from other trypanosomes. **(A)** Sequence alignment of MVKs from four related species. TeMVK gene was cloned and sequenced, and the protein sequence aligned to TbMVK (Q581L1, UniProt), TcMVK (Q4DJT8), and LmMVK (Q4Q6K7). Identical and similar residues are shown in black and gray, respectively. **(B)** *Ab initio* secondary structure prediction using Jpred4. TeMVK was aligned with LmMVK. Three conserved motifs 1, 2, and 3 (orange, red, and magenta bars) and residues known to bind mevalonate (asterisks) in LmMVK are indicated. Cylinders, α-helices; Arrows, β-sheet strands.

TeMVK is composed of 329 amino acids and has molecular weight 35.54 kDa. It has high sequence identity with *T*. *brucei* MVK (TbMVK; 98.8%), *T. cruzi* MVK (TcMVK; 63.6%) and *L. major* MVK (LmMVK; 60.2%) (Figure 1A). LmMVK is the only trypanosomatid MVK whose structure has been resolved by X-ray crystallography (Sgraja et al., 2007). An unbiased secondary structure prediction for TeMVK suggested an arrangement very similar to that of LmMVK (Figure 1B), with the N-terminal domain (residues 1–179) organized as seven β-sheet strands and four α-helices, and the C-terminal domain (residues 180–329) organized as five β-sheet strands and five α-helices. The primary sequence of TeMVK showed conservation of three motifs (referred as 1, 2, and 3) known to stabilize the conformation of the catalytic pocket, and all residues required for substrate interaction in LmMVK (Figure 1B).

**Figure 2:**
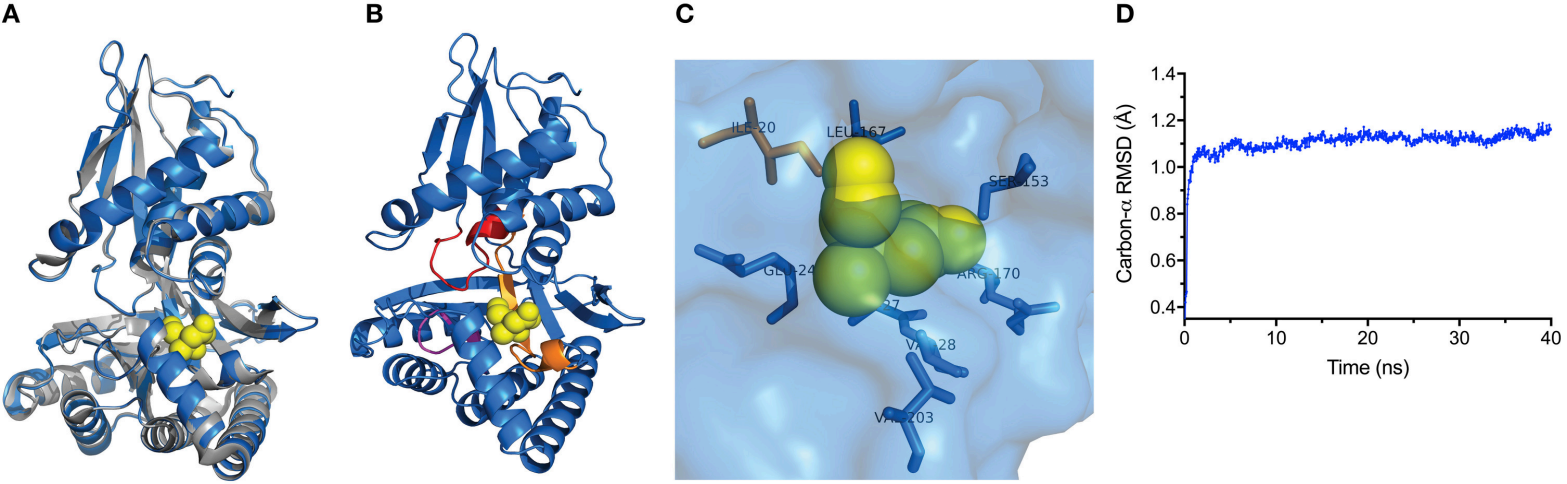
TeMVK homology model is structurally similar to *L. major* MVK. **(A)** Homology molecular modeling of TeMVK using the crystal structures of LmMVK (2HFU and 2HFS, PDB). TeMVK (blue) and LmMVK (gray) are superimposed and mevalonate molecule (yellow) indicated within the catalytic cleft. **(B)** Cartoon representation shows three motifs (orange, red and magenta) that assemble the deep catalytic cavity in TeMVK. **(C)** Structural arrangement of TeMVK residues known to interact with mevalonate in LmMVK. **(D)** Average RMSD for carbon-α (in angstroms) throughout the molecular dynamic simulation.

We next took advantage of the LmMVK structural data to predict the TeMVK structure using homology molecular modeling. Superimposition of TeMVK and LmMVK structures revealed a similar arrangement (Figure 2A) characteristic of GHMP kinases consisting of two domains (Timson, 2007). The TeMVK displayed a catalytic pocket disposed between the N- and C-terminal domains and formed by the three highly conserved motifs (Figure 2B), with structural conservation of all residues shown to interact with the substrate in LmMVK (Figure 2C). The molecular dynamic simulation demonstrated stable protein conformation, with RMSD 0.909 Å SD ± 0.052 Å for the backbone and 1.109 ± 0.065 Å for the carbon-α chain (Figure 2D). The homology model showed satisfactory stereochemical quality in a Ramachandran plot and limited protein flexibility (Supplementary Figures 1,2), respectively. The high conservation of motifs that form the catalytic cleft and residues interacting directly with the substrate suggests that (R)-mevalonate binds to TeMVK in a conformation similar to that of LmMVK.

### TeMVK is active in tetrameric oligomer form

**Figure 3:**
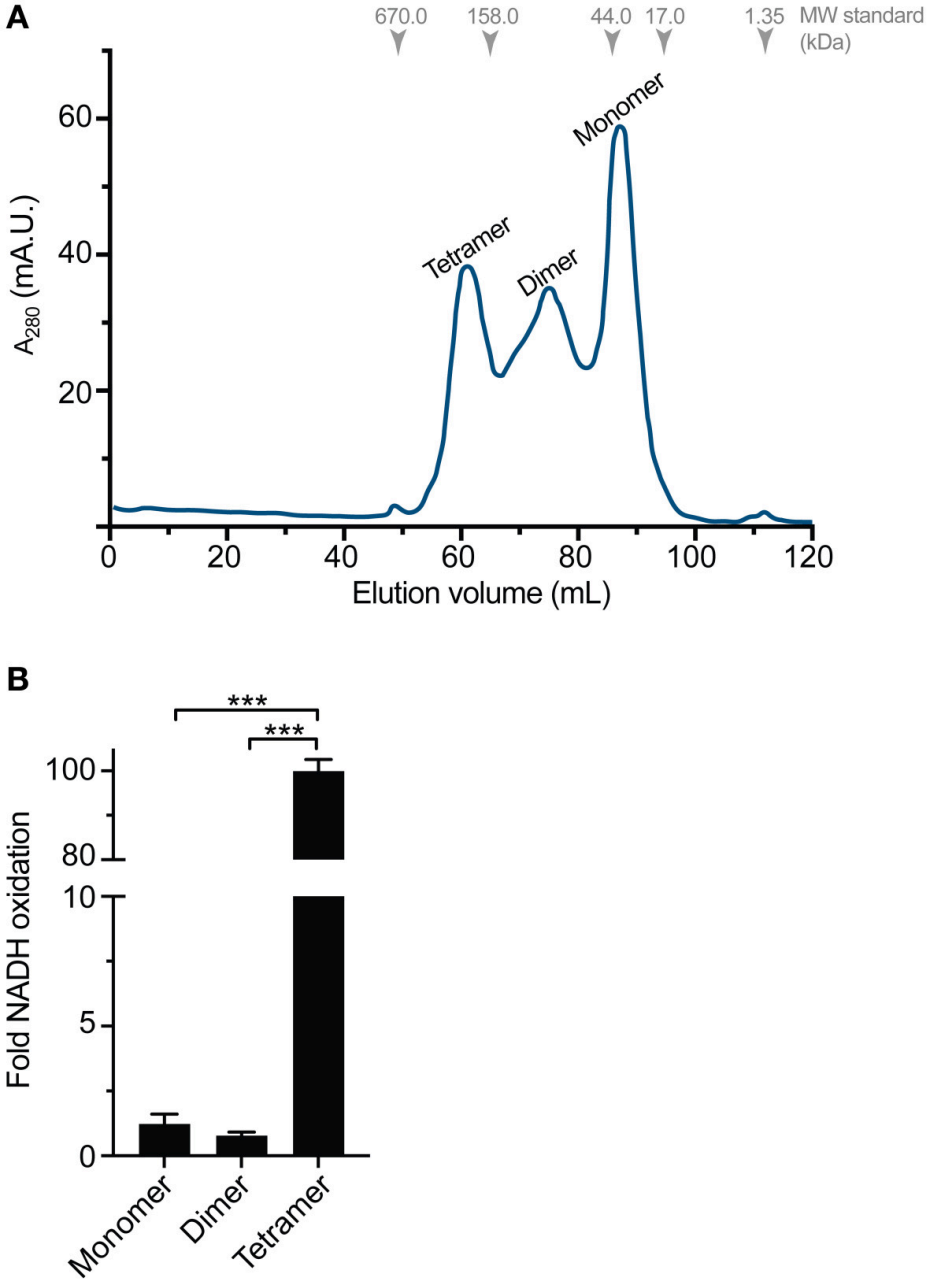
Distinct oligomeric states of recombinantly expressed TeMVK. **(A)** Gel filtration chromatogram (monitored at λ = 280 nm) of TeMVK expressed in autoinduction medium ZYM5052 and purified using a Superdex200 16/600 column in buffer containing 30 mM glycine, pH 9.0, and 150 mM NaCl. The three peaks correspond to distinct oligomers: tetramer, dimer, and monomer. **(B)** Fold increase of NADH oxidation using recombinantly expressed TeMVK. Bars indicate the mean ± SD from three independent experiments with three technical replicates (***P < 0.001, unpaired *t*-test).

Gel filtration chromatography from recombinant TeMVK expressed in autoinduction media revealed three peaks corresponding to distinct oligomeric states (Figure 3A) in comparison to recombinant protein expressed in LB media, which shows only a monomeric peak (Supplementary Figure 3). Size estimation calculation for the peaks corresponds to monomer, dimer and tetramer complexes of TeMVK.

Specific activity of the three different oligomeric states of purified TeMVK was evaluated using a fluorescence-based assay to measure NADH oxidation rate. Consumption rates were 1.35 × 10^−7^ (±0.3 × 10^−7^) moles NADH/min/mg for the monomer and 0.86 × 10^−7^ (±0.1 × 10^−7^) moles NADH/min/mg for the dimer. The consumption rate of the tetrameric oligomer was 1.1 × 10^−5^ (±2 × 10^−7^) moles NADH/min/mg, ~100-fold higher than the monomer and dimer (Figure 3B). Comparative specific activity values (BRENDA database) for MVKs from various organisms are shown in Table 1.

**Table 1:** Enzymatic activity of specific MVKs.

**Organism**	**Specific activity (μmol/min/mg)**	**Additional information**
*Leishmania major*	inactive	His-tagged purifed protein, monomer (Sgraja et al., 2007)
*Saccharomyces cerevisiae*	0.77	–
***Trypanosoma evansi***	**11.0**	**His-tagged purified protein, tetramer, pH 9.0**
*Staphylococcus aureus*	12.4	Purified enzyme, pH 7.5, 30°C
*Enterococcus faecalis*	24.0	Purified enzyme, pH 10, 37°C
*Rattus norvegicus*	32.7	His-tagged purified protein, pH 7.5, 25°C
*Homo sapiens*	37.0	pH 7.0, 30°C
*Trypanosoma cruzi*	72.8	His-tagged purified protein, dimer, pH 9.0 (Ferreira et al., 2016)
*Methanocaldococcus jannaschii*	387.0	–

*Specific activity values of recombinant MVKs previously described for various organisms. MVK activity from other organisms is generally observed with dimeric conformation (Fu et al., 2002). In contrast, TeMVK is active in its tetrameric conformation (bold, this study). Unless otherwise cited, this information was retrieved from BRENDA database (http://www.brenda-enzymes.info/)*.

### TeMVK is expressed *in Vivo* and colocalized with glycosomes

**Figure 4:**
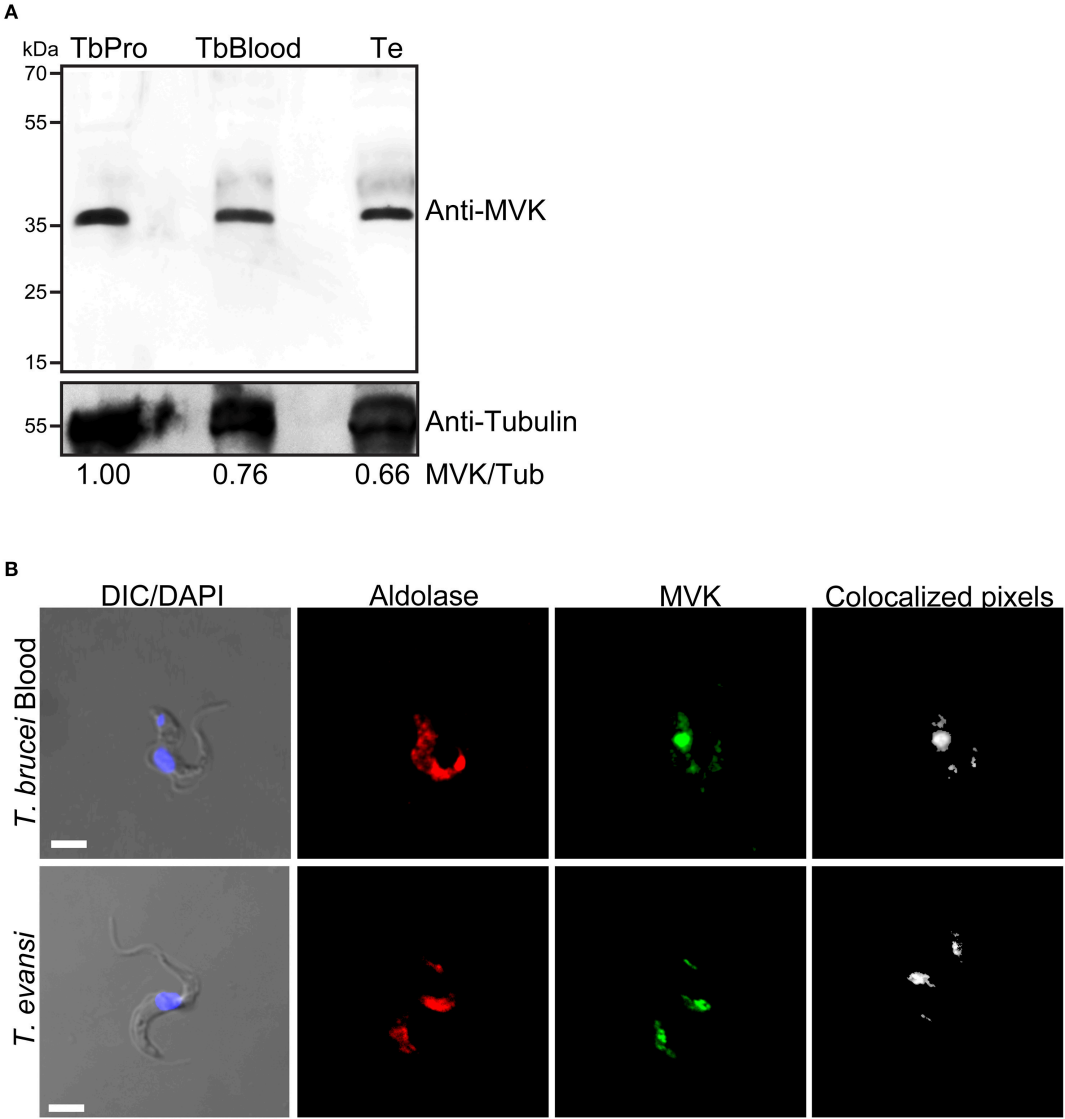
*T. evansi* expresses MVK (TeMVK) *in vivo*, and protein is localized in glycosomes. **(A)** Thirty micrograms of protein extracts of *T. brucei* procyclic form (TbPro), *T. brucei* bloodstream form (TbBlood), and *T. evansi* (Te) were subjected to SDS-PAGE and western blotting. Anti-TcMVK antibody raised in mice reveals expression in *T. evansi* and *T. brucei* of MVKs with similar molecular weight (~36 kDa). Anti-tubulin was used as loading control. Numbers refer to the densitometric ratio between TeMVK/tubulin. **(B)** Immunofluorescence using antibodies against aldolase (glycosomal marker; red) and MVK (green) reveals colocalization of the two enzymes in both *T.brucei* bloodstream form and *T.evansi*. Nuclei and kinetoplast were stained using DAPI. Images were obtained by confocal microscopy, and reconstructed using Imaris software program (Bitplane). Colocalized pixels are shown in white color. DIC, differential interference contrast. Scale bar: 3 μm.

*T. brucei* cells have glycosomes, peroxisome-related organelles that contain enzymes required for glycolysis (Opperdoes and Borst, 1977). MVK has been reported to colocalize with aldolase (a glycosomal marker enzyme) in both procyclic and bloodstream forms of *T. brucei* (Carrero-Lérida et al., 2009) and *T. cruzi* (Ferreira et al., 2016). The expression of MVK has never been reported in *T. evansi*. We aimed to verify whether *T. evansi* expresses TeMVK protein *in vivo* and identify its subcellular localization. Using a specific anti-TcMVK antibody (Ferreira et al., 2016), we demonstrated for the first time the presence of MVK in *T. evansi* cell extract from parasites isolated directly from murine blood (Figure 4A). TeMVK was colocalized with glycosomes, as revealed by immunofluorescence labeling of MVK and aldolase (Figure 4B).

### *T. evansi* expresses HMGCR protein

**Figure 5:**
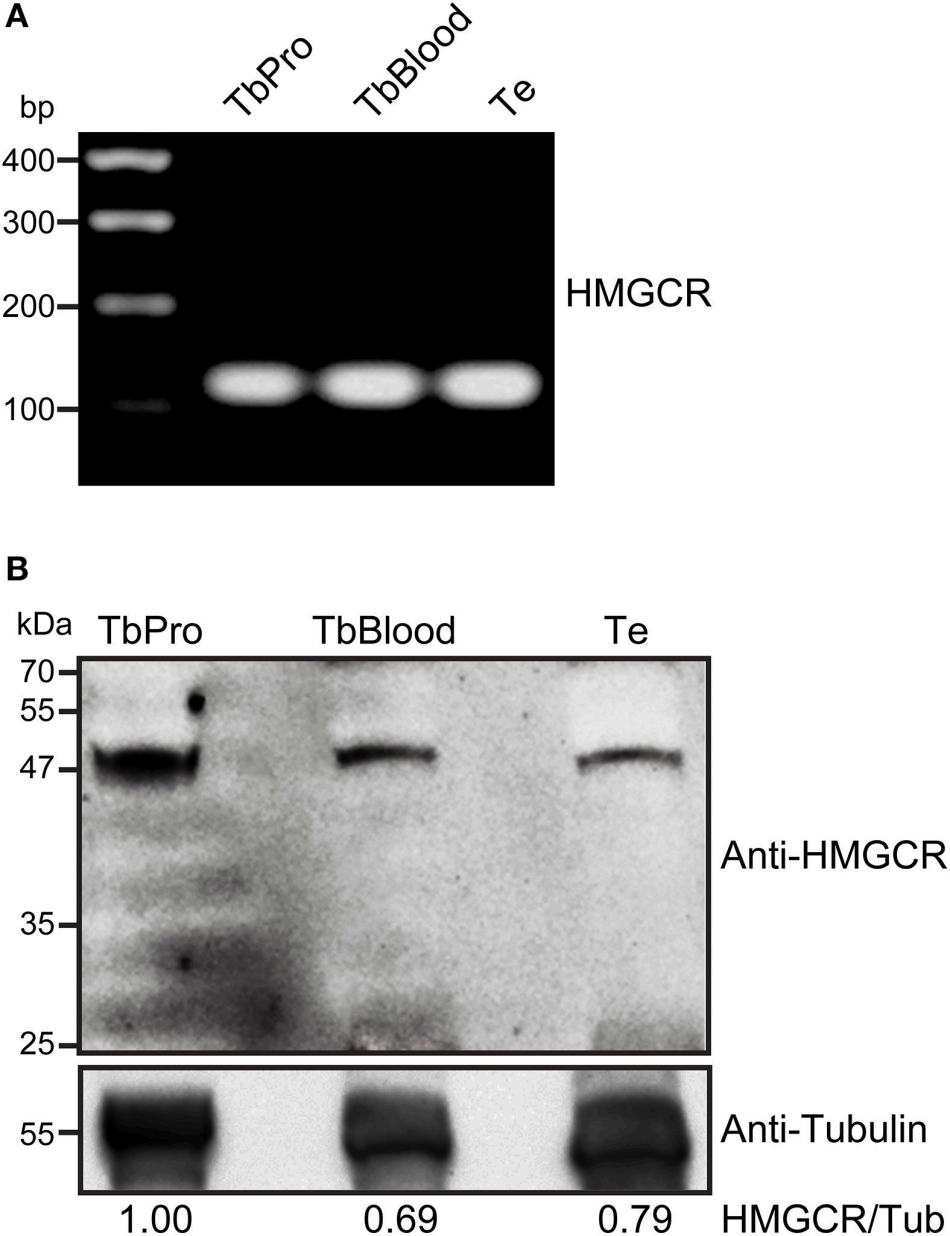
*T. evansi* expresses HMGCR at RNA and protein levels. **(A)** Presence of HMGCR mRNA in *T. evansi, T. brucei* procyclic form, and *T. brucei* bloodstream form, revealed by RT-PCR. HMGCR gene fragment contained 128 bp. Amplified fragments were sequenced and analyzed using DNASTAR Lasergene EditSeq for sequence confirmation. **(B)** Thirty micrograms of protein extracts of *T. brucei* procyclic form (TbPro), *T. brucei* bloodstream form (TbBlood) and *T. evansi* (Te) were subjected to SDS-PAGE and blotted using anti-human HMGCR. Anti-tubulin was used as loading control. Numbers refer to the densitometric ratio between HMGCR/tubulin.

We sought for other components of mevalonate pathway and identified the presence of HMGCR, an enzyme essential for synthesis of TeMVK substrate. HMGCR has been shown to localize to mitochondria in *L. major* and *T. cruzi* (Peña-Diaz et al., 2004). HMGCR transcripts were produced in both procyclic and bloodstream forms of *T. brucei* and *T. evansi* (Figure 5A). In addition, HMGCR was detected at the protein level in both species (Figure 5B). *T. brucei* bloodstream form and *T. evansi* showed comparable expression levels of HMGCR in the western blot. However, we were unable to verify the subcellular localization of HMGCR in *T.evansi* using commercial antibodies.

## Discussion

The isoprenoid biosynthesis pathway in the protozoan parasite *T. evansi* has not been examined previously. We confirmed the presence of the gatekeeper enzymes involved in this pathway in *T. evansi*, with focus on MVK. The *T. evansi* MVK (termed TeMVK) showed high sequence and structural similarity to MVK from other trypanosomes, notably *T. brucei* and *L. major*. TeMVK colocalized with glycosomes and was active *in vitro* only in its tetrameric oligomer form.

The presence of the three motifs assembling a catalytic site and residues known to coordinate substrate binding indicates a conserved mechanism for catalysis by MVKs in trypanosomatids. Sgraja et al. (2007) observed no activity for the monomeric form of recombinant TbMVK, which has 98.8% identity sequence identity with TeMVK. By contrast, TcMVK is active only in its dimeric form (Ferreira et al., 2016) as already demonstrated for mevalonate kinases from other species (Fu et al., 2002). Our findings suggest that the tetrameric (rather than monomeric or dimeric) form of MVKs from *T. evansi* and *T. brucei* displays catalytic activity *in vitro*. The methodology described here for expression and purification of active tetrameric oligomer form of TeMVK may be applicable to the expression of oligomeric states of recombinant proteins in other parasite species. Whether the tetrameric oligomer is the predominant active form of TeMVK *in vivo* remains to be determined. The presence of MVK in glycosomes from other trypanosomatids has been previously reported (Carrero-Lérida et al., 2009; Ferreira et al., 2016), supporting our finding that TeMVK is almost exclusively located in glycosomes.

The initial step in the mevalonate pathway is synthesis of mevalonate, catalyzed by HMGCR with HMG-CoA as substrate. HMGCR is a gatekeeper enzyme and is subject to several regulatory mechanisms (Sharpe and Brown, 2013). Its substrate conversion is a rate-limiting step in synthesis of cholesterol (ergosterol) and other isoprenoids, and occurs upstream of MVK activity. HMGCR is localized within mitochondria in both *L. major* and *T. cruzi* (Peña-Diaz et al., 2004), and almost exclusively in mitochondria of *T. brucei* procyclic form (Heise and Opperdoes, 2000). Although *T. evansi* lacks organized mitochondria (reviewed in Dean et al., 2013), it expresses HMGCR at mRNA and protein levels, as demonstrated in the present study. *T. brucei* procyclic form displays fully functional mitochondria and thus HMGCR may play a more important role at this stage.

The present findings demonstrate for the first time the presence of proteins required for isoprenoid biosynthesis in *T. evansi* isolated from infected animals. The involvement of mevalonate pathway in the synthesis of GPI required to tether VSGs on plasma membrane from African trypanosomatids (Smith and Bütikofer, 2010) provides new potential candidates for rational targeting. Additionally, compounds known to target this pathway (e.g., simvastatin and lovastatin; Urbina et al., 1993; Coppens et al., 1995) have shown anti-trypanosome activity and provide a hint that its inhibition could also show an effect against *T. evansi*. Whether MVKs are essential for survival of African trypanosomes locked in trypomastigote form remains to be investigated. *T. evansi* appears to be very similar to *T. brucei* bloodstream form; how these two species cause quite distinct diseases is another intriguing question for future study.

## Ethics statement

All procedures performed in studies involving animals were in accordance with the ethical standards of the institution or practice at which the studies were conducted CETEA-UDESC n°1.28.11

## Author contributions

DD, ÉF, EH, LM, and DB conception of the study. DD, ÉF, EH, LM, and DB designed the experiments. DD, ÉF, FL, FB, and MDG performed the experiments. DD, ÉF, EH, LM, and DB interpretation of the results and data analysis. EH, LM, and DB contributed with reagents, materials, and analysis tools. DD and DB wrote the manuscript. All authors read and approved the final manuscript.

### Conflict of interest statement

The authors declare that the research was conducted in the absence of any commercial or financial relationships that could be construed as a potential conflict of interest.

## Supplementary material

Supplementary Figure 1 Ramachandran plot for the TeMVK homology model was generated using the RAMPAGE tool. All residues are found within the limits for their respective favorable conformations.

Supplementary Figure 2 Heat map of RMSD per residue (in angstroms) for the TeMVK model indicates a limited flexibility of the protein throughout the molecular dynamic simulation.

Supplementary Figure 3 Gel filtration chromatogram (monitored at λ = 280 nm) of TeMVK expressed in LB medium and purified using a Superdex200 16/600 column in buffer containing 30 mM glycine, pH 9.0, and 150 mM NaCl. Using this protocol only the monomeric form of TeMVK was obtained.

## References

[B1] Birhanu H. Gebrehiwot T. Goddeeris B. M. Büscher P. Van Reet N. (2016). New *Trypanosoma evansi* Type B isolates from ethiopian dromedary camels. PLoS Negl. Trop. Dis. 10:e0004556. 10.1371/journal.pntd.0004556 27035661 PMC4818106

[B2] Bringaud F. Riviere L. Coustou V. (2006). Energy metabolism of trypanosomatids: adaptation to available carbon sources. Mol. Biochem. Parasitol. 149, 1–9. 10.1016/j.molbiopara.2006.03.017 16682088

[B3] Carnes J. Anupama A. Balmer O. Jackson A. Lewis M. Brown R. (2015). Genome and phylogenetic analyses of *Trypanosoma evansi* reveal extensive similarity to *T. brucei* and multiple independente origins for dyskinetoplasty. PLoS Negl. Trop. Dis. 8:e3404 10.1371/journal.pntd.0003404 PMC4288722 25568942

[B4] Carrero-Lérida J. Perez-Moreno G. Castillo-Acosta V. M. Ruiz-Perez L. M. Gonzalez-Pacanowska D. (2009). Intracellular location of the early steps of the isoprenoid biosynthetic pathway in the Trypanosomatids *Leishmania major* and *Trypanosoma brucei*. Int. J. Parasitol. 39, 307–314. 10.1016/j.ijpara.2008.08.012 18848949

[B5] Coppens I. Bastin P. Levade T. Courtoy P. J. (1995). Activity, pharmacological inhibition and biological regulation of 3-hydroxy-3-methylglutaryl coenzyme A reductase in *Trypanosoma brucei*. Mol. Biochem. Parasitol. 69, 29–40. 7723786 10.1016/0166-6851(94)00192-p

[B6] Coppens I. Courtoy P. J. (1996). The mevalonate pathway in parasitic protozoa and helminths. Exp. Parasitol. 82, 76–85. 10.1006/expr.1996.0011 8617335

[B7] Dean S. Gould M. K. Dewar C. E. Schnaufer A. C. (2013). Single point mutations in ATP synthase compensate for mitochondrial genome loss in trypanosomes. Proc. Natl. Acad. Sci. U.S.A. 110, 14741–14746. 10.1073/pnas.1305404110 23959897 PMC3767566

[B8] Desquesnes M. Dargantes I. Lai D.-H. Lun Z.-H. Holzmuller P. Jittapalapong S. (2013a). *Trypanosoma evansi* and surra: a review and perspectives on transmission, epidemiology and control, impact, and zoonotic aspects. BioMed Res. Int. 2013:321237 10.1155/2013/321237 24151595 PMC3789323

[B9] Desquesnes M. Holzmuller P. Lai D.-H. Dargantes I. Lun Z.-H. Jittapalapong S. (2013b). *Trypanosoma evansi* and surra: a review and perspectives on origin, history, distribution, taxonomy, morphology, hosts, and pathogenic effects. Biomed Res. Int. 2013:194176 10.1155/2013/194176 24024184 PMC3760267

[B10] Duarte D. P. Tavares K. C. S. Lazzarotto C. R. Komati L. K. O. Ferreira E. R. Bahia D. (2014). Genetic Profile of two isolates of *Trypanosoma evansi* from southern Brazil with different parasitaemias. Biotemas 27, 73–80. 10.5007/2175-7925.2014v27n3p73

[B11] Ferreira É. R. Horjales E. Bonfim-Melo A. Cortez C. da Silva C. V. De Groote M. (2016). Unique behavior of *Trypanosoma cruzi* mevalonate kinase, a conserved glycosomal enzyme involved in host cell invasion and signaling. Sci. Rep. 6:24610 10.1038/srep24610 27113535 PMC4845012

[B12] Fu Z. Wang M. Potter D. Miziorko H. M. Kim J. J. (2002). The structure of a binary complex between a mammalian mevalonate kinase and ATP: insights into the reaction mechanism and human inherited disease. J. Biol. Chem. 277, 18134–18142. 10.1074/jbc.M200912200 11877411

[B13] Grab D. J. Bwayo J. J. (1982). Isopycnic isolation of African trypanosomes on Percoll gradients formed *in situ*. Acta Trop. 39, 363–366. 6131595

[B14] Heise N. Opperdoes F. R. (2000). Localisation of a 3-Hydroxy-3-methylglutaryl-coenzyme a reductase in the mitochondrial matrix of *Trypanosoma brucei* procyclics. Z Naturforsch C. 55, 473–477. 10.1515/znc-2000-5-626 10928562

[B15] Hirumi H. Martin S. Hirumi K. Inoue N. Kanbara H. Saito A.. (1997). Cultivation of bloodstream forms of *Trypanosoma brucei* and *T. evansi* in a serum-free medium. Trop. Med. Int. Health 2, 240–244. 9491102 10.1046/j.1365-3156.1997.d01-268.x

[B16] Lai D. H. Hashimi H. Lun Z. R. Ayala F. J. Lukes J. (2008). Adaptations of *Trypanosoma brucei* to gradual loss of kinetoplast DNA: *Trypanosoma equiperdum* and *Trypanosoma evansi* are petite mutants of *T. brucei*. Proc. Natl. Acad. Sci. U.S.A. 105, 1999–2004. 10.1073/pnas.0711799105 18245376 PMC2538871

[B17] Lanham S. M. (1968). Separation of trypanosomes from the blood of infected rats and mice by anion-exchangers. Nature 218, 1273–1274. 5656665 10.1038/2181273a0

[B18] Lovell S. C. Davis I. W. Arendall W. B. III. de Bakker P. I. Word J. M. Prisant M. G.. (2003). Structure validation by Calpha geometry: phi, psi and Cbeta deviation. Proteins 50, 437–450. 10.1002/prot.10286 12557186

[B19] Opperdoes F. R. Borst P. (1977). Localization of nine glycolytic enzymes in a microbody-like organelle in *Trypanosoma brucei*: the glycosome. FEBS Lett. 80, 360–364. 10.1016/0014-5793(77)80476-6 142663

[B20] Peña-Diaz J. Montalvetti A. Flores C. L. Constán A. Hurtado-Guerrero R. De Souza W.. (2004). Mitochondrial localization of the mevalonate pathway enzyme 3-Hydroxy-3-methyl-glutaryl-CoA reductase in the Trypanosomatidae. Mol Biol Cell. 15:1356-63. 10.1091/mbc.E03-10-0720 14699057 PMC363142

[B21] Kumar R Singh J. Singh R. Kumar S. Yadav S. C. (2015). Comparative efficacy of different *in vitro* cultivation media for *Trypanosoma evansi* isolated from different mammalian hosts inhabiting different geographical areas of India. J. Parasit. Dis. 39, 174–178. 10.1007/s12639-013-0314-5 26063995 PMC4456538

[B22] Schnaufer A. Domingo G. J. Stuart K. D. (2002). Natural and induced dyskinetoplastid trypanosomatids: how to live without mitochondrial DNA. Int. J. Parasitol. 32, 1071–1084. 10.1016/S0020-7519(02)00020-6 12117490

[B23] Sgraja T. Smith T. K. Hunter W. N. (2007). Structure, substrate recognition and reactivity of *Leishmania major* mevalonate kinase. BMC Struct. Biol. 7:20. 10.1186/1472-6807-7-20 17397541 PMC1851959

[B24] Sharpe L. J. Brown A. J. (2013). Controlling cholesterol synthesis beyond 3-hydroxy-3-methylglutaryl-CoA reductase (HMGCR). J. Biol. Chem. 288, 18707–18715. 10.1074/jbc.R113.479808 23696639 PMC3696645

[B25] Silva R. A. M. S. Seidl A. Ramirez L. Dávila A. M. R. (2002). Trypanosoma Evansi e Trypanosoma Vivax: Biologia, Diagnóstico e Controle. Available online at: https://www.alice.cnptia.embrapa.br/bitstream/doc/810940/1/Livro015.pdf

[B26] Smith T. K. Bütikofer P. (2010). Lipid metabolism in *Trypanosoma brucei*. Mol. Biochem. Parasitol. 172, 66–79. 10.1016/j.molbiopara.2010.04.001 20382188 PMC3744938

[B27] Studier F. W. (2005). Protein production by auto-induction in high-density shaking cultures protein expression and purification. Protein Expr. Purif. 41, 207–234. 10.1016/j.pep.2005.01.016 15915565

[B28] Timms M. W. van Deursen F. J. Hendriks E. F. Matthews K. R. (2002). Mitochondrial development during life cycle differentiation of African trypanosomes: evidence for a kinetoplast-dependent differentiation control point. Mol. Biol. Cell 13, 3747–3759. 10.1091/mbc.E02-05-0266 12388771 PMC129980

[B29] Timson D. J. (2007). GHMP Kinases – Structures, mechanisms and potential for therapeutically relevant inhibition. Curr. Enzyme Inhibit. 3, 77–94. 10.2174/157340807779815431

[B30] Tuntasuvan D. Jarabrum W. Viseshakul N. Mohkaew K. Borisutsuwan S. Theeraphan A.. (2003). Chemotherapy of surra in horses and mules with diminazene aceturate. Vet. Parasitol. 110, 227–233. 10.1016/S0304-4017(02)00304-7 12482651

[B31] Uilenberg G. (1998). A Field Guide for the Diagnosis, Treatment and Prevention of African Animal Trypanosomosis. Rome: Food and Agriculture Organization of United Nations.

[B32] Urbina J. A. Lazardi K. Marchan E. Visbal G. Aguirre T. Piras M. M.. (1993). Mevinolin (lovastatin) potentiates the antiproliferative effects of ketoconazole and terbinafine against Trypanosoma (Schizotrypanum) cruzi: *in vitro* and *in vivo* studies. Antimicrob. Agents Chemother. 37, 580–591. 8460926 10.1128/aac.37.3.580 PMC187710

