# Supplementary figures and images for: Molecular Characterization of Trypanosoma evansi Mevalonate Kinase (TeMVK)
Source: Front Cell Infect Microbiol. 2018 Jul 10;8:223. doi: 10.3389/fcimb.2018.00223 (PMC6048237; doi:10.3389/fcimb.2018.00223)

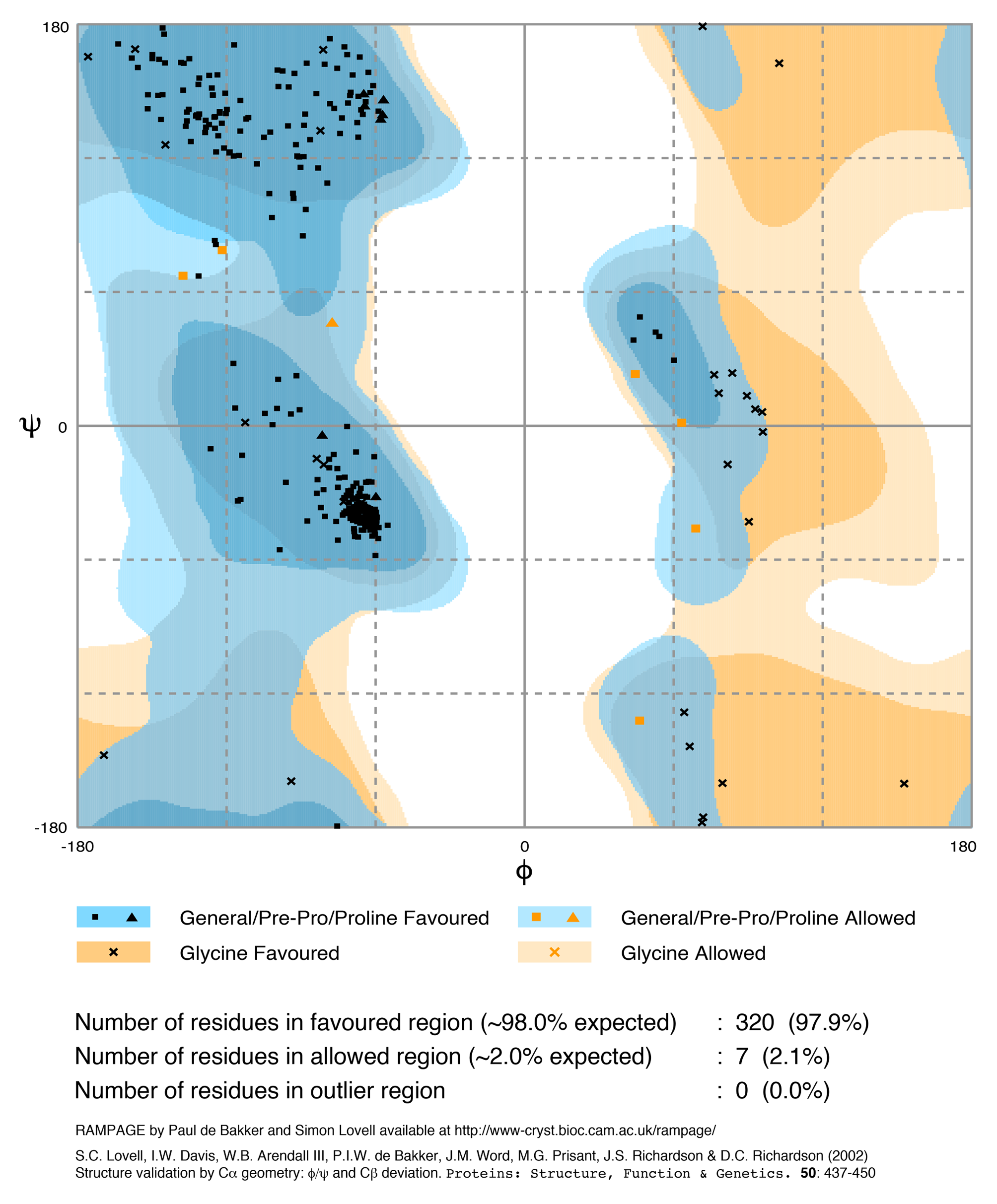

Supplement: Supplementary Figure 1 — Ramachandran plot for the TeMVK homology model was generated using the RAMPAGE tool. All residues are found within the limits for their respective favorable conformations. [file Image_1.TIF]

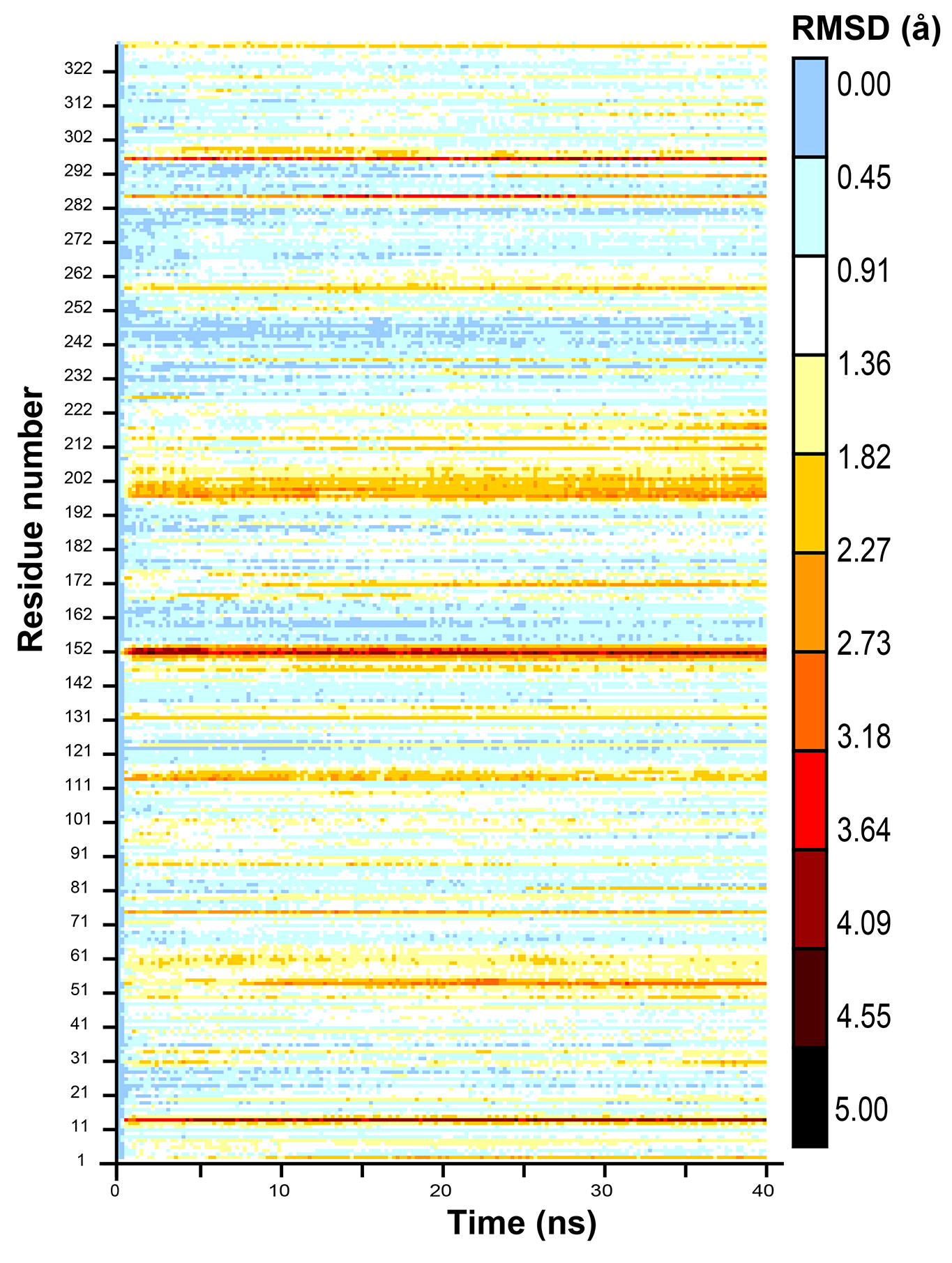

Supplement: Supplementary Figure 2 — Heat map of RMSD per residue (in angstroms) for the TeMVK model indicates a limited flexibility of the protein throughout the molecular dynamic simulation. [file Image_2.TIF]

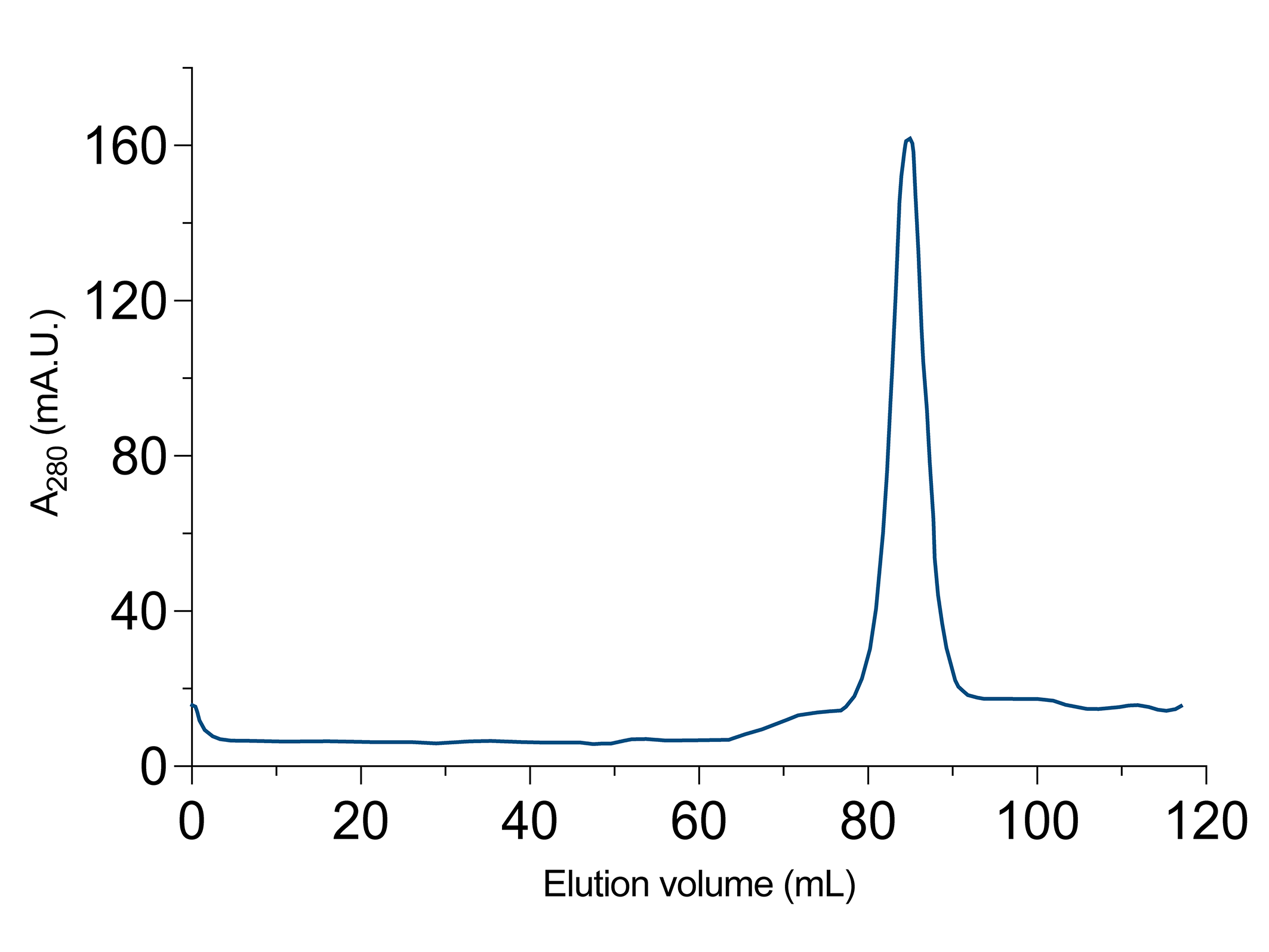

Supplement: Supplementary Figure 3 — Gel filtration chromatogram (monitored at λ = 280 nm) of TeMVK expressed in LB medium and purified using a Superdex200 16/600 column in buffer containing 30 mM glycine, pH 9.0, and 150 mM NaCl. Using this protocol only the monomeric form of TeMVK was obtained. [file Image_3.tif]
